# Translational shutdown and evasion of the innate immune response by SARS-CoV-2 NSP14 protein

**DOI:** 10.1073/pnas.2101161118

**Published:** 2021-05-27

**Authors:** Jack Chun-Chieh Hsu, Maudry Laurent-Rolle, Joanna B. Pawlak, Craig B. Wilen, Peter Cresswell

**Affiliations:** ^a^Department of Immunobiology, Yale University School of Medicine, New Haven, CT 06520;; ^b^Section of Infectious Diseases, Department of Internal Medicine, Yale University School of Medicine, New Haven, CT 06520;; ^c^Department of Laboratory Medicine, Yale School of Medicine, New Haven, CT 06520;; ^d^Yale Cancer Center, Yale School of Medicine, New Haven, CT 06520;; ^e^Department of Cell Biology, Yale University School of Medicine, New Haven, CT 06520

**Keywords:** NSP14, translation inhibition, coronavirus, immune evasion, innate immunity

## Abstract

The ongoing COVID-19 pandemic has caused an unprecedented global health crisis. Severe acute respiratory syndrome coronavirus 2 (SARS-CoV-2) is the causative agent of COVID-19. Subversion of host protein synthesis is a common strategy that pathogenic viruses use to replicate and propagate in their host. In this study, we show that SARS-CoV-2 is able to shut down host protein synthesis and that SARS-CoV-2 nonstructural protein NSP14 exerts this activity. We show that the translation inhibition activity of NSP14 is conserved in human coronaviruses. NSP14 is required for virus replication through contribution of its exoribonuclease (ExoN) and N7-methyltransferase (N7-MTase) activities. Mutations in the ExoN or N7-MTase active sites of SARS-CoV-2 NSP14 abolish its translation inhibition activity. In addition, we show that the formation of NSP14−NSP10 complex enhances translation inhibition executed by NSP14. Consequently, the translational shutdown by NSP14 abolishes the type I interferon (IFN-I)-dependent induction of interferon-stimulated genes (ISGs). Together, we find that SARS-CoV-2 shuts down host innate immune responses via a translation inhibitor, providing insights into the pathogenesis of SARS-CoV-2.

## Significance

To establish infection, pathogenic viruses have to overcome the type I interferon (IFN-I) antiviral response. A previous study demonstrated that the SARS-CoV-2 NSP14 is able to inhibit IFN-I responses. In this study, we report that SARS-CoV-2 NSP14 is a virus-encoded translation inhibitory factor which shuts down host protein synthesis, including synthesis of antiviral proteins. Our finding reveals a mechanism by which SARS-CoV-2 evades host antiviral responses. A comprehensive understanding of the strategies employed by SARS-CoV-2 to subvert host immune responses is critical for the design of next-generation antivirals and to prepare for future emerging viral pathogens.

Severe acute respiratory syndrome coronavirus 2 (SARS-CoV-2) is responsible for the ongoing COVID-19 pandemic that has caused more than 120 million confirmed cases, resulting in more than 2.7 million deaths globally (https://covid19.who.int). SARS-CoV-2 belongs to the Coronaviridae family, in the genus *Betacoronavirus*, which also includes two highly pathogenic human coronaviruses, severe acute respiratory syndrome coronavirus (SARS-CoV) and Middle East respiratory syndrome coronavirus (MERS-CoV) (1). These human coronaviruses are associated with severe lower respiratory tract infection leading to severe and fatal respiratory syndromes in humans. Currently, there is an urgent need to better understand the molecular mechanisms of SARS-CoV-2 pathogenesis, which will help us to design better antivirals and next-generation vaccines.

Replication of coronaviruses shuts down host protein synthesis in infected cells (2, 3). Multiple coronavirus proteins have been shown to hijack the host translation machinery to facilitate viral protein production. Despite the lack of sequence homology, coronavirus NSP1 proteins employ divergent mechanisms to suppress host protein expression (4). SARS-CoV NSP1 binds the small ribosomal subunit at the messenger RNA (mRNA) entry tunnel and inhibits mRNA translation (5, 6). Similarly, ribosome interaction and translation inhibition activity have been reported recently for SARS-CoV-2 NSP1 (7–10). Moreover, SARS-CoV accessory protein ORF7a and structural proteins spike (S) and nucleocapsid (N) proteins have been shown to inhibit protein synthesis (11–13). The precise mechanisms of translation inhibition by these SARS-CoV proteins remain to be determined. Given the protein homology between SARS-CoV and SARS-CoV-2, it is likely that multiple SARS-CoV-2 viral proteins harbor translational regulation activity.

SARS-CoV-2 has two large open reading frames (ORFs), ORF1a and ORF1b, encoding multiple nonstructural proteins (NSPs) involving every aspect of viral replication. ORF1a and ORF1b undergo proteolytic cleavage by viral-encoded proteinases to generate 16 mature NSPs, NSP1 to NSP16. Coronavirus NSP14 proteins are known to have 3′ to 5′ exoribonuclease (ExoN) activity and guanine-N7-methyltransferase activity (N7-MTase). The N-terminal ExoN domain is predicted to provide proofreading activity allowing removal of mismatched nucleotides introduced by the viral RNA-dependent RNA polymerase (14, 15). Given the large viral genome of coronaviruses, the proofreading activity of the ExoN domain is critical to maintain a high level of replication fidelity (16, 17). Recently, it has been shown that mutations in the active site and ZF motifs of the ExoN domain result in a lethal phenotype in SARS-CoV-2 and MERS-CoV (18). The C-terminal domain of NSP14 contains an S-adenosyl methionine (SAM)-dependent N7-MTase, which plays a critical role in viral RNA 5′ capping (19, 20). The 5′ cap facilitates viral mRNA stability and translation and prevents detection by host innate antiviral responses. SARS-CoV NSP14 forms a protein complex with NSP10, which is a zinc-binding protein with no reported enzymatic activity (20). NSP10 interacts with the N-terminal ExoN domain of NSP14 and enhances the ExoN activity but not the N7-MTase activity (14, 20). Notably, mutations in NSP10 that abolish the NSP14−NSP10 interaction result in a lethal phenotype in SARS-CoV (21).

In this study, we investigated the ability of SARS-CoV-2 NSP14 to suppress host protein synthesis and the type I interferon (IFN-I) response. Similar to SARS-CoV infection (2), we found that SARS-CoV-2 shuts down host protein synthesis. As shown for SARS-CoV (5, 6), and, more recently, for SARS CoV-2 (7–9), we confirmed that overexpression of NSP1 reduces protein synthesis in cells. In addition, we found that overexpression of NSP14 induces a near-complete shutdown in cellular protein synthesis. We also determined that the translation inhibition activity of NSP14 is conserved in several human coronaviruses. We demonstrated that mutations that inactivate either ExoN or N7-MTase enzymatic activities reverse translation inhibition mediated by NSP14. We also found that the formation of an NSP14−NSP10 protein complex enhances translation inhibition executed by NSP14 and showed that mutation of residues critical for this interaction abolishes this enhanced activity. Translation inhibition by NSP14 blocks IFN-I−dependent ISG induction, inhibiting the production of antiviral proteins. Our results provide mechanistic insights into the evasion of the innate immune responses by NSP14, a SARS-CoV-2 encoded translation inhibitor.

## Results

### SARS-CoV-2 Infection Induces Translational Shutdown.

**Fig. 1.**
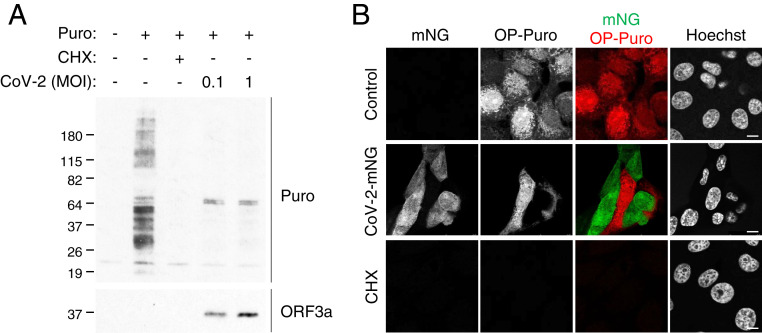
SARS-CoV-2 infection inhibits cellular translation. (*A*) Vero E6 cells were infected with SARS-CoV-2 at the indicated MOIs. After 24 h of infection, cells were pulse labeled with puromycin for 15 min. Puromycin incorporation was determined by immunoblotting using anti-puromycin antibody (Puro). Expression of SARS-CoV-2 viral protein was determined using anti-SARS-CoV ORF3a antibody (ORF3a). (*B*) Confocal images of Vero E6 cells infected with recombinant SARS-CoV-2 expressing mNeonGreen (CoV-2-mNG) (22) at an MOI of 0.5. After 24 h of infection, cells were pulse labeled with OP-Puro for 1 h, fixed, fluorescently labeled by the Click chemistry reaction, and stained by Hoechst. (Scale bars, 10 µm.)

Coronaviruses shut down host translation during viral replication (2, 3), and the newly emerged human coronavirus SARS-CoV-2 NSP1 protein was shown to inhibit translation (7–10). To further evaluate the effect of SARS-CoV-2 on host translation, we examined total protein synthesis during SARS-CoV-2 infection. Vero E6 cells were infected with SARS-CoV-2. At 24 h postinfection, total protein synthesis was measured by a puromycin incorporation assay. We observed that SARS-CoV-2 infection reduced protein synthesis (Fig. 1*A*). Notably, upon SARS-CoV-2 infection, two protein bands at ∼64 kDa, absent in uninfected cells and hence presumably viral proteins, were dominantly labeled by puromycin (Fig. 1*A*). These results suggest that SARS-CoV-2 infection shuts down cellular translation while promoting viral protein translation. We next examined whether the translational inhibition is restricted to the infected cells in the culture. Vero E6 cells were infected with recombinant SARS-CoV-2 expressing fluorescent protein mNeonGreen (CoV-2-mNG) (22). At 24 h postinfection, cellular protein synthesis was assessed by an O-propargyl puromycin (OP-Puro) labeling assay and confocal microscopy. We observed OP-Puro labeling in the uninfected cells, but not in the CoV-2-mNG−positive cells, suggesting that the protein synthesis inhibition is restricted to the SARS-CoV-2−infected cells (Fig. 1*B*).

### SARS-CoV-2 NSP14 Inhibits Cellular Translation.

**Fig. 2.**
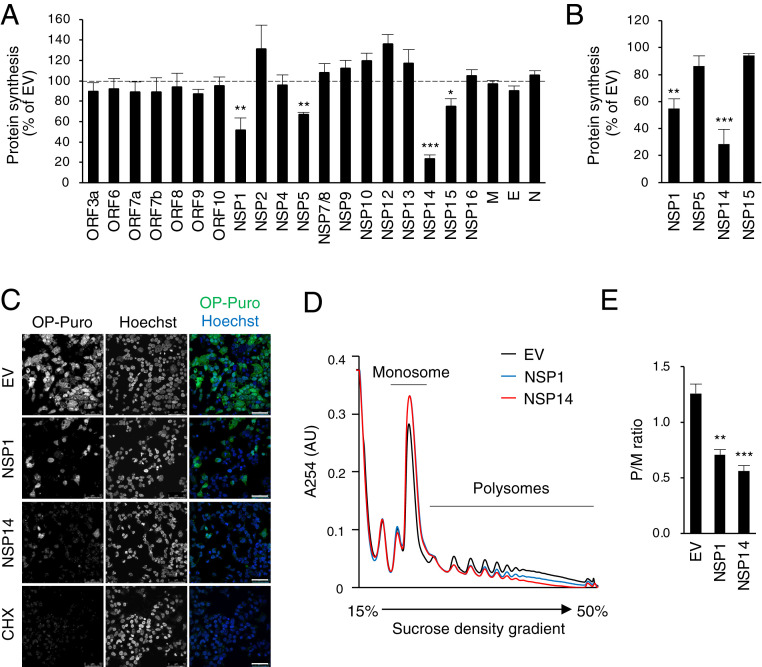
SARS-CoV-2 NSP14 inhibits cellular translation. (*A*) The 293T cells were transfected with plasmids encoding SARS-CoV-2 viral proteins. After 24 h of transfection, cells were pulse labeled with OP-Puro for 1 h, fixed, fluorescently labeled by the Click chemistry reaction, and analyzed by fluorescence-activated cell sorter (FACS). EV, empty vector. (*B*) The 293T cells were transfected with plasmids encoding indicated viral proteins for 24 h and puromycin labeled for 15 min. Puromycin incorporation was determined by immunoblotting using anti-puromycin antibody. (*C*) The 293T cells transfected and OP-Puro labeled as shown in *A*. Cells were stained by Hoechst and analyzed by confocal microscopy. (Scale bars, 50 µm.) (*D*) The 293T cells were transfected with plasmids encoding indicated viral proteins for 24 h. Cell lysates were cleared by centrifugation, loaded onto a 15 to 50% sucrose gradient, and subjected to ultracentrifugation. Absorbance was monitored at 254 nm to record the polysome profile. The monosome and polysome pools are indicated. (*E*) Quantification of polysome profile assay. Data are represented as the polysome-to-monosome (P/M) ratio. For *A*, *B*, and *E*, data are shown as mean ± SD of three biological repeats. *P < 0.05, **P < 0.01, ***P < 0.001 by unpaired Student’s *t* test.

It has been reported that SARS-CoV-2 NSP1 inhibits translation. However, overexpression of NSP1 in cells showed only moderate translation inhibition (∼50%) (7, 8). Given the difference in the efficiency of translation inhibition between SARS-CoV-2 infection (Fig. 1) and NSP1 overexpression, we considered the possibility that other viral proteins contributed to translational regulation. To investigate this, we examined translation in 293T cells overexpressing SARS-CoV-2 proteins, using OP-Puro labeling (Fig. 2*A*). The results showed that NSP1, NSP5, NSP14, and NSP15 significantly reduced OP-Puro labeling, suggesting that multiple SARS-CoV-2 proteins are involved in translation inhibition. Consistent with the previous studies (7, 8), we showed that NSP1 inhibited translation in 293T cells by ∼50% (Fig. 2*A*). However, overexpression of NSP14 reduced OP-Puro labeling even more significantly, by ∼75% (Fig. 2*A*), suggesting that NSP14 is a potent translation inhibitor. Inhibition was also apparent by a puromycin incorporation assay and by confocal microscopy of the OP-Puro−labeled 293T cells (Fig. 2 *B* and *C*). We further confirmed the translation inhibition activity of NSP14 by polysome profiling using sucrose density gradient centrifugation. Compared with empty vector control, the polysome profiles showed a drastic decrease in translating polyribosomes and an increase in 80S ribosomes in the presence of NSP14 (Fig. 2*D*, red line), indicating global inhibition of translation. Consistent with a previous study, NSP1 induced a similar shift (8), although this was less pronounced than that observed with NSP14 (Fig. 2*D*, blue line). These decreases in translation efficiency were quantified by the ratio of actively translating polysomes versus monosomes (P/M ratio), showing that NSP14 significantly inhibits cellular translation (Fig. 2*E*).

SARS-CoV-2 NSP1 inhibits nuclear export of host mRNA by poly(A) RNA nuclear retention and degradation in the cytoplasm (23). To determine whether NSP14 regulates mRNA stability and nuclear export, we performed RNA fluorescence in situ hybridization (FISH) to detect poly(A) RNA in 293T cells (*SI Appendix*, Fig. S1). The results showed that NSP14 has no effect on the poly(A) RNA distribution between the nucleus and cytoplasm (*SI Appendix*, Fig. S1*A*), nor on the total intracellular poly(A) RNA level (*SI Appendix*, Fig. S1*B*). To further examine whether NSP14 inhibits translation by inhibiting transcription, we coexpressed NSP14 with an EGFP reporter plasmid. Consistent with the results that measured global translation, we observed a significant reduction in EGFP fluorescence, quantified by flow cytometry, in the presence of NSP14, but not in the presence of NSP10 (*SI Appendix*, Fig. S2*A*). No significant change in the *EGFP* mRNA level was detected by qRT-PCR in the presence of either NSP14 or NSP10 (*SI Appendix*, Fig. S2*B*). Together, these results provide evidence that the translation inhibition by NSP14 is not a result of mRNA degradation or unpaired nuclear export.

### Human Coronavirus NSP14 Proteins Inhibit Cellular Translation.

**Fig. 3.**
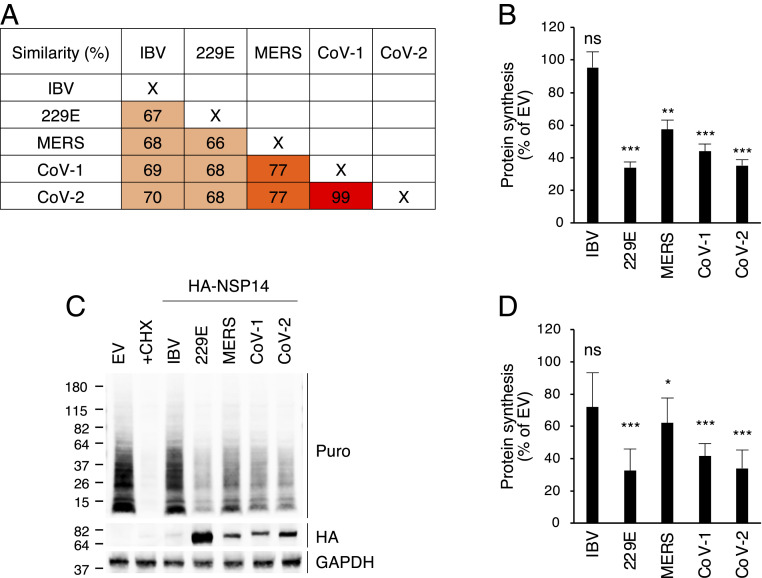
Human coronavirus NSP14 proteins inhibit cellular translation. (*A*) Comparison of the amino acid sequences of coronavirus NSP14. Sequence similarity of NSP14 between avian gammacoronavirus (IBV), human alphacoronavirus (HCoV-229E; 229E), and human betacoronaviruses (MERS-CoV; MERS, SARS-CoV; CoV-1, SARS-CoV-2; CoV-2). (*B*) The 293T cells were transfected with plasmids encoding different HA-tagged NSP14. After 24 h of transfection, cells were pulse labeled with OP-Puro for 1 h, fixed, fluorescently labeled by the Click chemistry reaction, and analyzed by FACS. (*C*) The 293T cells were transfected for 24 h and puromycin labeled for 15 min. Puromycin incorporation was determined by immunoblotting using anti-puromycin antibody (Puro). HA-tagged NSP14 proteins were detected by anti-HA antibody (HA). (*D*) Quantification of puromycin incorporation assay shown in *C*. For *B* and *D*, data are shown as mean ± SD of three biological repeats. *P < 0.05, **P < 0.01, ***P < 0.001 by unpaired Student’s *t* test; ns, not significant.

NSP14 is a highly conserved viral protein in coronaviruses. In human betacoronaviruses, NSP14 of SARS-CoV-2 exhibits 99% and 77% amino acid sequence similarity to that of SARS-CoV and MERS-CoV, respectively (Fig. 3*A*). NSP14 of SARS-CoV-2 also shows ∼70% amino acid sequence similarity to NSP14 of HCoV-229E (human alphacoronavirus) and infectious bronchitis virus (IBV; an avian gammacoronavirus) (Fig. 3*A*). Given this homology, we asked whether NSP14 proteins of different coronaviruses could mediate translation inhibition. We determined cellular translation activity using OP-Puro labeling of 293T cells overexpressing HA-tagged NSP14 proteins from different coronaviruses. The results showed that all three human betacoronavirus NSP14 proteins inhibited translation (Fig. 3*B*). Moreover, alphacoronavirus HCoV-229E NSP14 exhibited a similar degree of translation inhibition, whereas IBV NSP14 only slightly reduced translation (Fig. 3*B*). This result was replicated using a puromycin incorporation assay (Fig. 3 *C* and *D*). Notably, transfection of equal amounts of plasmid DNA resulted in a much lower IBV NSP14 protein level than that of human coronaviruses (Fig. 3*C*). Therefore, 293T cells were transfected with increasing amounts of plasmid DNA and subjected to a puromycin incorporation assay. We found that IBV NSP14 was poorly expressed even with high amounts of plasmid DNA transfection, which likely resulted in weak translation inhibition (*SI Appendix*, Fig. S3). In contrast, transfection of the human coronavirus NSP14 constructs resulted in a dose-dependent inhibition of puromycin incorporation (*SI Appendix*, Fig. S3). These results suggest that the NSP14 proteins of human coronaviruses share a critical role in translation inhibition during coronavirus infection.

### Mutations Reducing ExoN and N7-MTase Activities Reverse Translation Inhibition.

**Fig. 4.**
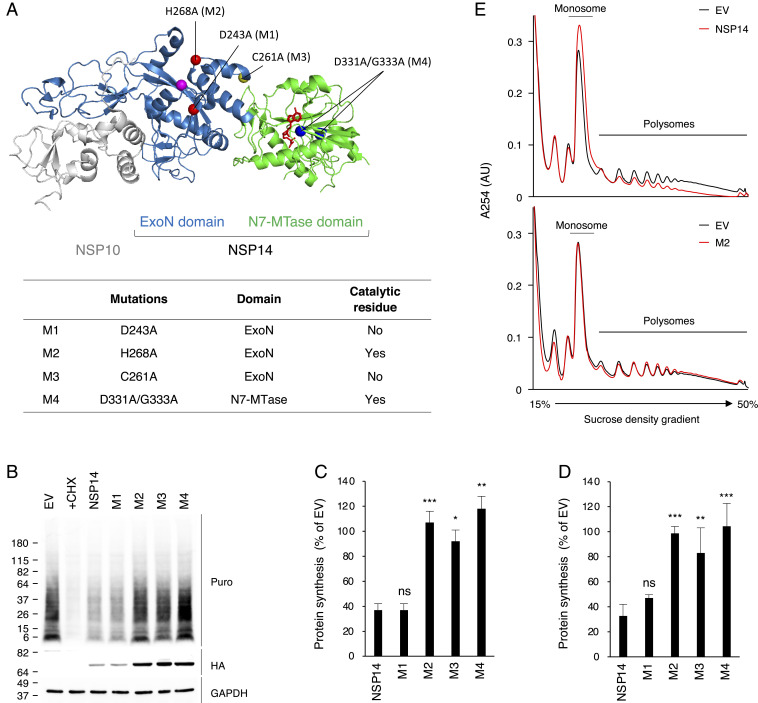
ExoN and N7-MTase are required for translation inhibition activity of NSP14. (*A*) Crystal structure of SARS-CoV NSP10−NSP14 complex (Protein Data Bank ID code 5NFY). NSP10 is shown in gray. N-terminal ExoN and C-terminal N7-MTase domains are in blue and green, respectively. NSP14 mutants are described below, in the table. The mutation residues are highlighted in the structure. (*B*) The 293T cells were transfected with plasmids encoding WT or NSP14 mutants for 24 h and puromycin labeled for 15 min. Puromycin incorporation was determined by immunoblotting using anti-puromycin antibody (Puro). HA-tagged NSP14 proteins were detected by anti-HA antibody (HA). (*C*) Quantification of puromycin incorporation assay shown in *B*. (*D*) The 293T cells were transfected with plasmids encoding WT or NSP14 mutants. After 24 h of transfection, cells were pulse labeled with OP-Puro for 1 h, fixed, fluorescently labeled, and analyzed by FACS. (*E*) The 293T cells were transfected with plasmids encoding NSP14 or M2 mutant for 24 h. Cell lysates were cleared by centrifugation, loaded onto a 15 to 50% sucrose gradient, and subjected to ultracentrifugation. Absorbance was monitored at 254 nm to record the polysome profile. The monosome and polysome pools are indicated. For *C* and *D*, data are shown as mean ± SD of three biological repeats. *P < 0.05, **P < 0.01, ***P < 0.001 by unpaired Student’s *t* test.

NSP14 is a member of the superfamily of DEDDh exoribonucleases, which contain five conserved active-site residues critical for ExoN activity (15, 20, 24). Additionally, there are two zinc finger (ZF) motifs, which contribute to the structural stability and catalytic activity of the ExoN domain (20). To determine whether the ExoN activity of SARS-CoV-2 NSP14 is responsible for translation inhibition, we generated a catalytically inactive NSP14 mutant, H268A (M2) (Fig. 4*A*). Using OP-Puro labeling and puromycin incorporation assays, we showed that H268A mutation (M2) in the ExoN active site abolished the translation inhibition activity (Fig. 4 *B*–*D*). These results were confirmed by polysome profiling in 293T cells (Fig. 4*E*). We then generated a ZF 2 motif mutant, C261A (M3) that structurally destabilizes the ExoN activity (20). We found that, although the C261A mutation is distal from the active site, M3 failed to inhibit translation (Fig. 4 *B*–*D*). In contrast, the mutant M1, with an alanine substitution at the noncatalytic D243 residue, D243A, retained the translation inhibition activity (Fig. 4 *B*–*D*). These results suggest that a functional ExoN domain is required to inhibit translation. Finally, we asked whether the N7-MTase activity is required. We found that D331A/G333A double mutation (M4) in the N7-MTase active site (20) abolished the translational inhibition activity of NSP14 (Fig. 4 *B*–*D*), suggesting that the N7-MTase activity is also required for translation inhibition. Notably, transfection of equal amounts of plasmids encoding NSP14 and its mutants resulted in much lower NSP14 and M1 protein levels than the other mutants (Fig. 4*B*). However, no such reduction was found on the NSP14 mRNA level (*SI Appendix*, Fig. S2*C*). This indicates that NSP14 inhibits not only cellular protein synthesis but its own mRNA translation, which has been shown in overexpression of SARS-CoV and SARS-CoV-2 NSP1 (6–8). Together, these data are consistent with the suggestion that both the ExoN and N7-MTase activities of SARS-CoV-2 NSP14 are required to inhibit translation.

### NSP10 Enhances Translation Inhibition Activity of NSP14.

**Fig. 5.**
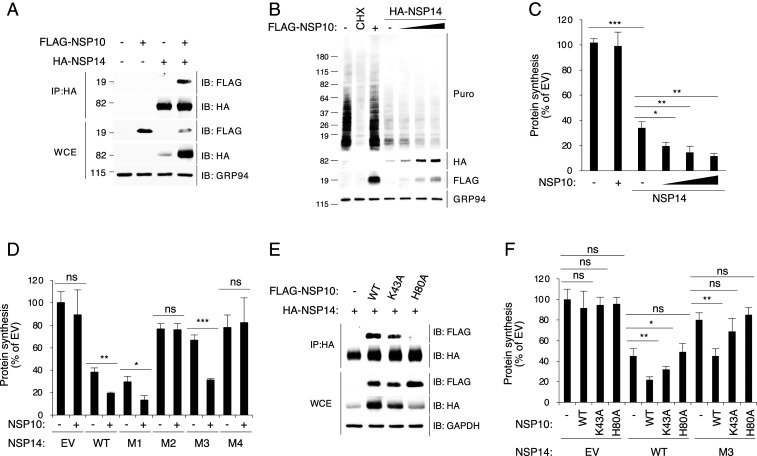
NSP14−NSP10 complex formation enhances the translation inhibition activity of NSP14. (*A*) The 293T cells were transfected with plasmids encoding indicated proteins for 24 h. Cell lysates were subjected to IP using mouse anti-HA antibody and followed by immunoblotting with rabbit anti-HA and anti-FLAG antibodies. WCE, whole cell extract. (*B*) The 293T cells were transfected with plasmids encoding NSP10 or NSP14 for 24 h and puromycin labeled for 15 min. Puromycin incorporation was determined by immunoblotting using anti-puromycin antibody (Puro). HA-tagged NSP14 and FLAG-tagged NSP10 proteins were detected by anti-HA and anti-FLAG antibodies, respectively. (*C*) Quantification of puromycin incorporation assay shown in *B*. (*D*) The 293T cells were transfected for 24 h and puromycin labeled for 15 min. Puromycin incorporation was determined by immunoblotting. (*E*) The 293T cells were cotransfected with HA-tagged NSP14 and FLAG-tagged NSP10 or its mutants for 24 h. Cell lysates were subjected to IP using mouse anti-HA antibody and followed by immunoblotting with rabbit anti-HA and anti-FLAG antibodies. (*F*) The 293T cells were transfected for 24 h and puromycin labeled for 15 min. Puromycin incorporation was determined by immunoblotting. For *C*, *D*, and *F*, data are shown as mean ± SD of three biological repeats. *P < 0.05, **P < 0.01, ***P < 0.001 by unpaired Student’s *t* test.

SARS-CoV NSP14 forms a protein complex with NSP10 (Fig. 4*A*) (20). NSP10 is a central modulator in the replication and transcription complex of coronaviruses (21). Moreover, NSP10 activates the enzymatic activity of NSP14 and NSP16 through protein−protein interaction (20, 21). NSP10 interacts with the N-terminal ExoN domain of NSP14, selectively enhancing the ExoN activity but not the N7-MTase activity (14, 25). Given the high protein sequence homology between SARS-CoV and SARS-CoV-2 (*SI Appendix*, Figs. S4 and S5), we hypothesized that SARS-CoV-2 NSP10 and NSP14 might also interact with each other. Immunoprecipitation (IP) experiments involving 293T cells expressing both NSP10 and NSP14 revealed that the two proteins coprecipitated (Fig. 5*A*). We then examined whether NSP10 interaction affects the translation inhibition activity of NSP14. Using the puromycin incorporation assay, we showed that, although NSP14 inhibited translation as expected, coexpression of NSP10 further enhanced the inhibition (Fig. 5 *B* and *C*). Notably, NSP10 coexpression significantly increased the NSP14 protein level (Fig. 5*B*), consistent with the idea that the formation of the complex increases NSP14 protein stability and consequently enhances the observed inhibition of translation. Next, we investigated whether the complex formation can rescue the translation inhibition activity of NSP14 mutants. Using puromycin incorporation assays, we found that coexpression of NSP10 with wild-type (WT) NSP14 and the mutants increased the translation inhibition activity of WT NSP14 and the active mutant M1 (Fig. 5*D* and *SI Appendix*, Fig. S6*A*). Notably, we found that the translation inhibition activity of M3, known to have a structurally destabilized N-terminal ExoN domain in SARS-CoV NSP14, was restored by coexpression of NSP10 (Fig. 5*D* and *SI Appendix*, Fig. S6*A*). In contrast, coexpression with NSP10 failed to rescue the translation inhibition activities of M2 and M4 bearing mutations in the active sites (Fig. 5*D* and *SI Appendix*, Fig. S6*A*). These results suggest that coexpression with NSP10 enhances the translation inhibition activity of NSP14 primarily by association and structural stabilization.

Several residues in the SARS-CoV NSP14−NSP10 interface have been reported to be required for the protein−protein interaction and activation of the ExoN activity of NSP14, including the amino acid residues Lys43 and His80 of NSP10 (21). We therefore generated two SARS-CoV-2 NSP10 mutants bearing alanine substitutions at these residues (K43A and H80A) (*SI Appendix*, Fig. S6 *B* and *C*). Consistent with the SARS-CoV study (21), IP assays showed that NSP10 (K43A) partially reduced the NSP10−NSP14 interaction, whereas NSP10 (H80A) completely lost the NSP14−NSP10 interaction (Fig. 5*E*). Consequently, as shown by the puromycin incorporation assay, coexpression of WT NSP14 with NSP10 (H80A) mutant did not enhance the translation inhibition, whereas NSP10 (K43A) mutant induced a moderate increase in translation inhibition (Fig. 5*F* and *SI Appendix*, Fig. S6*D*). Moreover, we found that the NSP10 mutants failed to enhance the translation inhibition activity of the structurally destabilized NSP14 mutant M3 (Fig. 5*F* and *SI Appendix*, Fig. S6*D*). Together, the results suggest that NSP10−NSP14 interaction enhances the translation inhibition activity.

### NSP14 Inhibits IFN-Dependent ISG Induction.

**Fig. 6.**
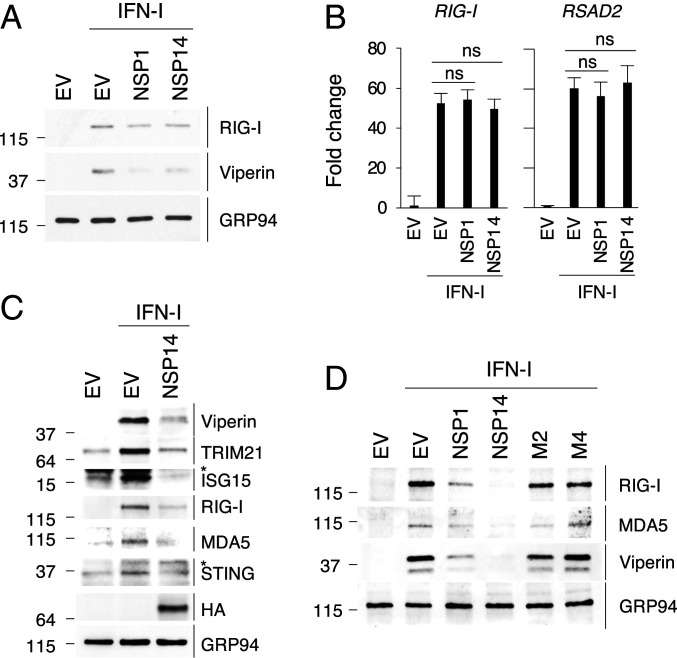
NSP14 inhibits the protein expression of ISGs. (*A*) Vero E6 cells were transfected with plasmids encoding NSP1 or NSP14 for 24 h and treated with IFN-I for 18 h. The expression of IFN-stimulated proteins was determined by immunoblotting with antibodies against depicted proteins. (*B*) The induction of ISGs on mRNA level from *A* were determined by qRT-PCR with primers against genes encoding RIG-I and viperin proteins (*RSAD2*). (*C*) The 293T cells were transfected with HA-tagged NSP14 for 24 h and treated with IFN-I for 18 h. The induction of ISGs was determined by immunoblotting with antibodies against depicted proteins. Asterisk (*) indicates nonspecific protein bands. (*D*) The 293T cells were transfected with plasmids encoding NSP1 or NSP14 for 24 h and treated with IFN-I for 18 h. The expression of IFN-stimulated proteins of ISGs was determined by immunoblotting with antibodies against depicted proteins. For *B*, data are shown as mean ± SD of six biological repeats by unpaired Student’s *t* test.

SARS-CoV-2 NSP14 overexpression inhibits the production of IFN-beta and IFN-stimulated genes (ISGs) (26–28). Consistent with these studies, we showed, by immunoblot analysis, that SARS-CoV-2 NSP14 overexpression suppresses endogenous ISG stimulation in response to IFN-I in Vero E6 cells (Fig. 6*A*). In contrast, NSP14 did not affect the induction of ISGs (Fig. 6*B*). As reported in the literature (8), NSP1 also inhibited ISG protein synthesis but not the mRNA (Fig. 6 *A* and *B*). This phenotype was examined for additional ISGs in 293T cells (Fig. 6*C*). Consistently, the results showed that NSP14 strongly inhibits the protein synthesis of antiviral ISGs (viperin, TRIM21, ISG15) and ISGs functioning in RNA sensing and signaling (retinoic acid-inducible gene I [RIG-I], MDA5, STING) in the IFN-I response (Fig. 6*C*). A recent preprint reported that, although SARS-CoV-2 infection up-regulates many ISGs on the mRNA level, their translation is impaired (29). Therefore, to determine whether the translation inhibition activity of NSP14 is responsible for inhibiting ISG expression, we evaluated the effect of the NSP14 mutants on the expression of endogenous ISGs in 293T cells. We found that M2 and M4 failed to inhibit the expression of ISGs, suggesting that inhibition of endogenous ISG expression by NSP14 is a result of its translation inhibition activity (Fig. 6*D*).

## Discussion

Similar to other coronaviruses (4), SARS-CoV-2 NSP1 was found to inhibit translation in in vitro translation systems by blocking the mRNA entry tunnel of the small ribosomal subunit, but it only exhibits moderate translation inhibition activity in cells (7, 8). K164A/H165A double mutations abolish the host gene suppression activities of NSP1 (7, 8). However, recombinant SARS-CoV bearing the mutations in NSP1 only partially inhibits the translation inhibition effect during viral replication (2). Moreover, the SARS-CoV mutant shows no difference in viral replication compared to WT virus (2). These observations suggest that multiple SARS-CoV-2 proteins may contribute to a translation shutdown. Here, using nascent peptide labeling assays, we showed that SARS-CoV-2 infection dramatically shuts down host translation (Fig. 1). Screening identified SARS-CoV-2 NSP14 as a novel inhibitor of host translation (Fig. 2). Using polysome profiling and nascent protein labeling coupled with flow cytometry and confocal microscopy, we found that NSP14 induces a more drastic reduction in host protein synthesis than NSP1 (Fig. 2). Polysome profiling in the presence of NSP14 exhibited a typical translation inhibition profile with an increase in 80S ribosome population and decrease in polysomes (Fig. 2 *D* and *E*). We also examined whether NSP14 regulates mRNA stability or nuclear export, using RNA FISH to detect poly(A) RNA. In cells expressing NSP14, unlike those expressing NSP1, we observed no effect on total intracellular poly(A) RNA levels, or on the poly(A) RNA distribution between the nucleus and cytoplasm, supporting a direct role for NSP14 in translation inhibition (*SI Appendix*, Fig. S1). Future experiments to determine the effect of NSP14-mediated translation inhibition on viral replication will require the generation of an appropriate recombinant virus.

Coronaviruses consist of four genera: alpha, beta, gamma, and delta. Human coronaviruses HCoV-229E and HCoV-NL63 belong to the alphacoronavirus genus, and HCoV-HKU1, HCoV-OC43, SARS-CoV, MERS-CoV, and SARS-CoV-2 belong to the betacoronavirus genus (1). Coronavirus NSP14 is a bifunctional enzyme, with both ExoN and N7-MTase activities requiring highly conserved residues in each active site. We showed that NSP14 proteins of four human coronaviruses, including three highly pathogenic betacoronaviruses, are able to inhibit host protein synthesis, which suggests that this role of NSP14 is conserved among these coronaviruses. While NSP14 proteins of HCoV-229E, SARS-CoV, and SARS-CoV-2 dramatically inhibit translation, MERS-CoV NSP14 translation inhibition was less pronounced (Fig. 3 *C* and *D*). In contrast, despite the high protein homology with human coronaviruses (Fig. 3*A*), NSP14 of the avian gammacoronavirus IBV only slightly inhibits translation (Fig. 3 *C* and *D*). Given that IBV is a host-specific coronavirus (30), we speculate that this relatively weak inhibition may result from low IBV NSP14 expression in human cells (*SI Appendix*, Fig. S13) and perhaps the lack of host-specific cofactors (30). Together, these observations probably reflect a critical role of translation inhibition during the replication of human coronaviruses.

Identification of NSP14 as a translation inhibitory protein was unexpected given that it has been characterized as a bifunctional enzyme. The N-terminal domain is a member of the superfamily of DEDDh ExoNs, which contains five conserved catalytic residues required for ExoN activity (15, 20, 24). These residues are essential for viral replication, as recombinant coronaviruses bearing ExoN active-site mutations are nonviable (18, 24). In this study, we investigated the role of the ExoN activity in translation inhibition by alanine substitution at residues D243 and H268 in the ExoN domain. Both residues were first reported as catalytic residues (24), whereas the SARS-CoV NSP14 crystal structure revealed that, despite being in close proximity, D243 is not a catalytic residue (20). While mutations at D243 and H268 abolish the ExoN activity of recombinant SARS-CoV and MERS-CoV NSP14 proteins (18–21), we found that the catalytic H268A mutation (M2) in the active site abolishes the translation inhibition activity, but D243A mutation (M1) has no effect (Fig. 4). Nevertheless, the H268A mutation result suggests a critical role for the ExoN domain in translation inhibition.

The ExoN domain has two ZF motifs, which contribute to the structural stability and catalytic activity of ExoN domain (20). Recombinant SARS-CoV NSP14 proteins bearing mutations in the ZF motifs abolish the ExoN activity, and similar mutations impair MERS-CoV replication (18, 20). Here, we found that C261A (M3), mutated in the ZF2 motif, does not inhibit translation (Fig. 4). Notably, the catalytic ExoN mutation H268A (M2) is in the ZF2 motif. These results suggest an important role of the ZF2 motif in translation inhibition activity of NSP14. Intriguingly, coexpression with NSP10 rescues the translation inhibition activity of M3 mutant (Fig. 5*D*). However, it has been shown that C261A mutation abolishes the ExoN activity even in the presence of NSP10 (20). This suggests that the structural integrity of the ExoN domain is essential for translation inhibition in a way that discriminates this activity from the ExoN activity.

Mutations in the ExoN and N7-MTase domains attenuate the in vitro RNA degradation and methyltransferase activity, respectively (14, 19, 31). Moreover, mutations in the ExoN domain do not interfere with the N7-MTase activity, and vice versa (18, 19, 31). The C-terminal N7-MTase domain is composed of a canonical SAM binding motif I that is responsible for viral RNA 5′ cap formation (19, 20). In vitro biochemical assays showed that mutations in the SAM binding motif of SARS-CoV and MERS-CoV NSP14 proteins result in inactivation of the N7-MTase activity (18–20, 31). Moreover, N7-MTase mutation impairs the replication of betacoronavirus murine hepatitis virus and results in delayed kinetics of viral mRNA translation in vitro (32). Here, we found that the N7-MTase mutant (M4) bearing a D331A/G333A double mutation in the SAM binding motif fails to inhibit translation (Fig. 4). Thus we have identified mutations in both ExoN and N7-MTase domains of SARS-CoV-2 NSP14 that abolish the translation inhibition activity, suggesting that both domains may function cooperatively to execute translation inhibition. It remains to be determined exactly how these domains combine to contribute to translation inhibition.

NSP10 is a central modulator in the coronavirus replication and transcription complex but has no reported specific enzymatic activity itself. It activates the enzymatic activity of both NSP14 and NSP16 by protein−protein interaction (20, 21). NSP10 interacts with the N-terminal ExoN domain of SARS-CoV NSP14 and strongly enhances the ExoN activity but not the N7-MTase activity in vitro (14, 25). Notably, mutations in NSP10 that abolish the NSP10−NSP14 interaction inactivate SARS-CoV (21). We show here that SARS-CoV-2 NSP10 also forms a protein complex with NSP14 (Fig. 5*A*). While this interaction is not required for NSP14-mediated translation inhibition, it substantially stabilizes NSP14 expression, and, as a consequence, translation inhibition is significantly enhanced (Fig. 5 and *SI Appendix*, Fig. S4).

The IFN-I response is the first line of defense against viral invasion, leading to the production of hundreds of ISGs, many of which play an important role in the antiviral responses.

Coronavirus infections are sensed by a cytosolic pattern recognition receptor, RIG-I, which activates this defense system (33, 34). Replication of human betacoronaviruses, including both SARS-CoV and SARS-CoV-2, is sensitive to IFN-I pretreatment (35, 36), and multiple betacoronavirus proteins have been shown to antagonize IFN-I production and downstream antiviral ISG expression (37–39). Recently, three independent studies using luciferase reporter assays identified multiple SARS-CoV-2 proteins as IFN-I antagonists, including NSP14 (26–28). A recent unreviewed preprint reported that, although SARS-CoV-2 infection dramatically up-regulates numerous ISGs, their translation is impaired (29). Consistently, our results indicate that NSP14 shuts down the expression of endogenous antiviral ISGs via its translation inhibition activity (Fig. 6). Both the ExoN and N7-MTase activities of NSP14 play critical roles in counteracting the IFN-I responses during the replication of coronaviruses (32, 40), and, consistent with this, we found that mutations in the ExoN and N7-MTase domains abolish inhibition of ISG induction (Fig. 6*D*). Although the translation of ISGs can be selectively attenuated by dengue virus-2 infection (41), SARS-CoV-2 NSP1 inhibits the synthesis of IFN-stimulated proteins by global translation inhibition (8). In addition to NSP1, we found that SARS-CoV-2 NSP14 inhibits the protein expression of a variety of ISGs via its global translation inhibition activity (Fig. 6*C*), which provides an additional layer of protection against the IFN response. This redundancy of ISG antagonism has been reported for coronaviruses (28, 42).

It remains to be determined how SARS-CoV-2 overcomes the translation shutdown induced by the NSPs for the production of its own viral proteins. We speculate that, as shown for SARS-CoV, a common sequence/structure in the 5′ untranslated region of viral mRNAs facilitates efficient translation of viral mRNAs (43). Nevertheless, this work provides evidence that the likely mechanism underlying the involvement of NSP14 in immune evasion lies in its capacity to interfere with host protein generation. Thus, NSP14 both ensures the fidelity of viral mRNA translation and blocks the translation of host mRNAs. Furthermore, the mutagenesis study of NSP14 enzymatic activity and NSP10–NSP14 interaction demonstrates several unique niches that may provide a starting point for development of potent antiviral drugs.

## Materials and Methods

### Constructs.

SARS-CoV-2 viral protein constructs were obtained from Adolfo García-Sastre (The Icahn School of Medicine at Mount Sinai, New York, NY). NSP14 mutants were generated from pCAGGS-HA-NSP14 using site-directed mutagenesis. IBV, HCoV-229E, MERS-CoV, and SARS-CoV NSP14 genes with HA tag were synthesized by Twist Bioscience and subcloned into pCAGGS vector. NSP10 mutants were generated from pCAGGS-FLAG-NSP10 using site-directed mutagenesis. All primers used for molecular cloning and qRT-PCR were synthesized by the Keck facility at Yale University. Plasmids were purified using Zymo Research kits.

### Antibodies.

Primary antibodies used were mouse anti-HA (901502, Biolegend), rabbit anti-HA (51064-2-AP, Proteintech), anti-FLAG (F7425, Sigma), anti-puromycin (12D10, Sigma), anti-viperin (MaP.VIP) (44), anti-RIG-I (3743, Cell Signaling Technology), anti-MDA5 (5321, Cell Signaling Technology), anti-TRIM21 (12108-1-AP, Proteintech), anti-ISG15 (200401438, Rockland), anti-STING (13647, Cell Signaling Technology), anti-GRP94 (9G10, Enzo), and anti-GAPDH (10494-1-AP, Proteintech). Anti-SARS-CoV ORF3a was a kind gift from Carolyn Machamer (Johns Hopkins University, Baltimore, MD). All secondary antibodies used for immunofluorescence imaging and immunoblotting were purchased from Invitrogen and Jackson ImmunoResearch, respectively.

### Cells.

The 293T and Vero E6 cells were grown in Dulbecco’s modified Eagle’s medium (DMEM; Gibco) supplemented with 10% fetal bovine serum (FBS; HyClone). For DNA plasmid transfection, cells were plated 1 d before and transfected using Lipofectamine 2000 according to the manufacturer’s instructions. For ISG induction, Vero E6 and 293T cells were treated with 1,000 u/mL and 2,000 U/mL, respectively, of Universal Type I IFN (PBL) for 18 h.

### Viruses and Cell Culture Infections.

High-titer stocks of SARS-CoV-2 virus (isolate USA-WA1/2020, CoV-2 WA1) obtained from BEI reagent repository and SARS-CoV-2-mNG (22) were obtained by passage in Vero E6 cells. Viral titers were determined by plaque assay on Vero E6 cells. Virus was cultured exclusively in a biosafety level 3 facility. Vero E6 cells were either mock infected or infected at the indicated multiplicity of infection (MOI) in serum-free DMEM for 1 h. Unbound virus was removed, and cells were maintained in 2% FBS/DMEM at 37 °C for 24 h. Viral replication was measured by plaque assay on Vero E6 cells.

### Puromycin Incorporation Assay.

Cells were infected or transfected for 24 h and incubated with 10 μg/mL of puromycin (Invivogen) in 10% FBS/DMEM for 15 min. Cells were harvested in Laemmli sample buffer supplement with 2-mercaptoethanol and analyzed by immunoblotting. Translation was estimated using ImageJ and normalized to empty vector controls. For cycloheximide (CHX) treatment, 50 µg/mL CHX was added 15 min before puromycin incorporation.

### OP-Puro Labeling Assay.

Cells were incubated with 50 μM OP-Puro in 10% FBS/DMEM for 1 h. Cells were harvested and washed with ice-cold PBS. Cells were fixed with 1% paraformaldehyde in PBS for 15 min on ice, washed in PBS, then permeabilized in permeabilization buffer (3% FBS and 0.1% saponin in PBS) for 5 min at room temperature (RT). The Click chemistry reaction was performed by incubating cells with Click solution (1 mM CuSO_4_, 5 μM azide conjugated Alexa Fluor 488, 150 mM Tris, pH 8.5, and 100 mM ascorbic acid added before use) for 30 min at RT. The cells were washed three times in permeabilization buffer and resuspended in PBS for flow cytometry analysis or confocal imaging.

### Polysome Profiling.

Cells were treated with 50 µg/mL CHX at 37 °C for 10 min, washed with ice-cold PBS, incubated with 50 µg/mL CHX/PBS at 4 °C for 20 min, and lysed by adding polysome lysis buffer (200 mM KOAc, 50 mM Tris, pH 7.4, 10 mM MgCl_2_, 1 mM dithiothreitol, 0.5% IGEPAL CA-630, RNaseOUT, and protease inhibitor mixture [Sigma]) on ice for 15 min. Cell lysates were centrifuged at 14,000 rpm for 15 min to remove cell debris. Polyribosomes were resolved on 15 to 50% linear sucrose gradients as previously described (45).

### Immunoblotting.

Cells were lysed in Laemmli sample buffer and boiled. Cell lysates were separated by sodium dodecyl sulfate polyacrylamide gel electrophoresis with 12% homemade gel or 4 to 15% gradient gel (Bio-Rad) and transferred onto poly(vinylidene difluoride) membranes (Millipore). Membranes were blocked in 5% bovine serum albumin (BSA)/Tris-buffered saline with 0.1% Tween 20 (TBST) for 1 h at RT, incubated with primary antibodies in 2% BSA/TBST overnight at 4 °C, incubated with secondary horseradish peroxidase-conjugated antibodies in 2% BSA/TBST for 1 h at RT, and analyzed by a ChemiDoc MP Imaging System (Bio-Rad).

### Confocal Immunofluorescence Microscopy.

Cells were washed with PBS and fixed with 4% paraformaldehyde in PBS for 15 min at RT. Cells were permeabilized with 0.1% Triton X-100 in PBS for 5 min at RT and blocked with 10% normal goat serum in PBST (0.2% Tween-20 in PBS). Primary and secondary stainings were performed in 2.5% normal goat serum/PBST. Primary antibodies used were the same as used for immunoblotting. Secondary antibodies were the Alexa Fluor series from Thermo Fisher Scientific. Slides were mounted with ProLong Gold Anti-Fade Reagent (Thermo Fisher Scientific), imaged by Leica SP8 model confocal microscope, and analyzed using ImageJ.

### Poly(A) RNA FISH.

Cells were rinsed with PBS and fixed with 4% paraformaldehyde in PBS for 10 min at RT and fixed with ice-cold methanol for 10 min. Cells were washed with 70% ethanol for 10 min and 1 M Tris⋅HCl, pH 8.0, for 5 min. Poly(A) RNA was stained in Hybridization Buffer (1mg/mL yeast transfer RNA, 0.005% BSA, 10% dextran sulfate, 25% formamide, deionized, in 2× saline-sodium citrate (SSC) buffer) supplemented with 1 ng/mL Cy5-oligo(dT)30 for 4 h at 37 °C. Cells were washed by 4× SSC buffer once and 2× SSC buffer twice. Primary and secondary stainings were performed in 2× SSC buffer supplemented with 0.1% triton-X100. Rabbit anti-HA antibody (Proteintech) was used to detect HA-tagged viral proteins. Secondary antibodies were the Alexa Fluor series from Thermo Fisher Scientific. Slides were mounted with ProLong Gold Anti-Fade Reagent (Thermo Fisher Scientific), imaged by Leica SP8 model confocal microscope, and analyzed using ImageJ.

### Immunoprecipitation.

The 293T cells were seeded in 60-mm dishes and transfected with the indicated plasmids or with an empty vector control for 24 h. Cells were lysed in IP buffer (20 mM Tris, pH 8, 150 mM NaCl, 1% Nonidet P-40, 5 mM MgCl_2_) supplemented with Protease Inhibitor Mixture (Sigma). Cell lysates were incubated with prewashed anti-HA magnetic beads (Thermo Fisher Scientific) for 2 h at 4 °C. Beads were washed extensively with TBST, and eluates were analyzed by immunoblotting as described above.

### qRT-PCR.

RNA was extracted from cells using TRIzol or AccuLift (Fluidigm) according to the manufacturers’ instructions. For one-step qRT-PCR, RNA was quantified using Luna Universal One-Step RT-qPCR Kit (NEB). Analyses were performed using an Mx3000P (Stratagene).

### Flow Cytometry.

Cells were harvested, washed with PBS, and collected using an Accuri C6 CSampler (BD Biosciences); >10,000 live cells were measured for each sample. Analysis was performed using FlowJo software.

### Statistical analyses.

Results from all studies were compared with unpaired two-tailed Student’s *t* test. *P* values less than 0.05 were considered significant.

## Supplementary Material

Supplementary File

## Data Availability

All study data are included in the article and *SI Appendix*.

## References

[1] Coronaviridae Study Group of the International Committee on Taxonomy of Viruses, The species severe acute respiratory syndrome-related coronavirus: Classifying 2019-nCoV and naming it SARS-CoV-2. Nat. Microbiol. 5, 536–544 (2020). 32123347 10.1038/s41564-020-0695-z PMC7095448

[2] K. Narayanan ., Severe acute respiratory syndrome coronavirus nsp1 suppresses host gene expression, including that of type I interferon, in infected cells. J. Virol. 82, 4471–4479 (2008). 18305050 10.1128/JVI.02472-07 PMC2293030

[3] A. Hilton, L. Mizzen, G. MacIntyre, S. Cheley, R. Anderson, Translational control in murine hepatitis virus infection. J. Gen. Virol. 67, 923–932 (1986). 3009691 10.1099/0022-1317-67-5-923

[4] K. Narayanan, S. I. Ramirez, K. G. Lokugamage, S. Makino, Coronavirus nonstructural protein 1: Common and distinct functions in the regulation of host and viral gene expression. Virus Res. 202, 89–100 (2015). 25432065 10.1016/j.virusres.2014.11.019 PMC4444399

[5] W. Kamitani, C. Huang, K. Narayanan, K. G. Lokugamage, S. Makino, A two-pronged strategy to suppress host protein synthesis by SARS coronavirus Nsp1 protein. Nat. Struct. Mol. Biol. 16, 1134–1140 (2009). 19838190 10.1038/nsmb.1680 PMC2784181

[6] K. G. Lokugamage, K. Narayanan, C. Huang, S. Makino, Severe acute respiratory syndrome coronavirus protein nsp1 is a novel eukaryotic translation inhibitor that represses multiple steps of translation initiation. J. Virol. 86, 13598–13608 (2012). 23035226 10.1128/JVI.01958-12 PMC3503042

[7] K. Schubert ., SARS-CoV-2 Nsp1 binds the ribosomal mRNA channel to inhibit translation. Nat. Struct. Mol. Biol. 27, 959–966 (2020). 32908316 10.1038/s41594-020-0511-8

[8] M. Thoms ., Structural basis for translational shutdown and immune evasion by the Nsp1 protein of SARS-CoV-2. Science 369, 1249–1255 (2020). 32680882 10.1126/science.abc8665 PMC7402621

[9] S. Yuan ., Nonstructural protein 1 of SARS-CoV-2 is a potent pathogenicity factor redirecting host protein synthesis machinery toward viral RNA. Mol. Cell 80, 1055–1066.e6 (2020). 33188728 10.1016/j.molcel.2020.10.034 PMC7833686

[10] C. P. Lapointe ., Dynamic competition between SARS-CoV-2 NSP1 and mRNA on the human ribosome inhibits translation initiation. Proc. Natl. Acad. Sci. U.S.A. 118, e2017715118 (2021). 33479166 10.1073/pnas.2017715118 PMC8017934

[11] S. A. Kopecky-Bromberg, L. Martinez-Sobrido, P. Palese, 7a protein of severe acute respiratory syndrome coronavirus inhibits cellular protein synthesis and activates p38 mitogen-activated protein kinase. J. Virol. 80, 785–793 (2006). 16378980 10.1128/JVI.80.2.785-793.2006 PMC1346853

[12] H. Xiao, L. H. Xu, Y. Yamada, D. X. Liu, Coronavirus spike protein inhibits host cell translation by interaction with eIF3f. PLoS One 3, e1494 (2008). 18231581 10.1371/journal.pone.0001494 PMC2204050

[13] B. Zhou ., The nucleocapsid protein of severe acute respiratory syndrome coronavirus inhibits cell cytokinesis and proliferation by interacting with translation elongation factor 1alpha. J. Virol. 82, 6962–6971 (2008). 18448518 10.1128/JVI.00133-08 PMC2446950

[14] M. Bouvet ., RNA 3′-end mismatch excision by the severe acute respiratory syndrome coronavirus nonstructural protein nsp10/nsp14 exoribonuclease complex. Proc. Natl. Acad. Sci. U.S.A. 109, 9372–9377 (2012). 22635272 10.1073/pnas.1201130109 PMC3386072

[15] F. Ferron ., Structural and molecular basis of mismatch correction and ribavirin excision from coronavirus RNA. Proc. Natl. Acad. Sci. U.S.A. 115, E162–E171 (2018). 29279395 10.1073/pnas.1718806115 PMC5777078

[16] L. D. Eckerle ., Infidelity of SARS-CoV Nsp14-exonuclease mutant virus replication is revealed by complete genome sequencing. PLoS Pathog. 6, e1000896 (2010). 20463816 10.1371/journal.ppat.1000896 PMC2865531

[17] L. D. Eckerle, X. Lu, S. M. Sperry, L. Choi, M. R. Denison, High fidelity of murine hepatitis virus replication is decreased in nsp14 exoribonuclease mutants. J. Virol. 81, 12135–12144 (2007). 17804504 10.1128/JVI.01296-07 PMC2169014

[18] N. S. Ogando ., The enzymatic activity of the nsp14 exoribonuclease is critical for replication of MERS-CoV and SARS-CoV-2. J. Virol. 94, e01246-20 (2020). 10.1128/JVI.01246-20 PMC7654266 32938769

[19] Y. Chen ., Functional screen reveals SARS coronavirus nonstructural protein nsp14 as a novel cap N7 methyltransferase. Proc. Natl. Acad. Sci. U.S.A. 106, 3484–3489 (2009). 19208801 10.1073/pnas.0808790106 PMC2651275

[20] Y. Ma ., Structural basis and functional analysis of the SARS coronavirus nsp14−nsp10 complex. Proc. Natl. Acad. Sci. U.S.A. 112, 9436–9441 (2015). 26159422 10.1073/pnas.1508686112 PMC4522806

[21] M. Bouvet ., Coronavirus Nsp10, a critical co-factor for activation of multiple replicative enzymes. J. Biol. Chem. 289, 25783–25796 (2014). 25074927 10.1074/jbc.M114.577353 PMC4162180

[22] X. Xie ., An infectious cDNA clone of SARS-CoV-2. Cell Host Microbe 27, 841–848.e3 (2020). 32289263 10.1016/j.chom.2020.04.004 PMC7153529

[23] K. Zhang ., Nsp1 protein of SARS-CoV-2 disrupts the mRNA export machinery to inhibit host gene expression. Sci. Adv. 7, eabe7386 (2021). 33547084 10.1126/sciadv.abe7386 PMC7864571

[24] E. Minskaia ., Discovery of an RNA virus 3′→5′ exoribonuclease that is critically involved in coronavirus RNA synthesis. Proc. Natl. Acad. Sci. U.S.A. 103, 5108–5113 (2006). 16549795 10.1073/pnas.0508200103 PMC1458802

[25] M. Bouvet ., In vitro reconstitution of SARS-coronavirus mRNA cap methylation. PLoS Pathog. 6, e1000863 (2010). 20421945 10.1371/journal.ppat.1000863 PMC2858705

[26] C. K. Yuen ., SARS-CoV-2 nsp13, nsp14, nsp15 and orf6 function as potent interferon antagonists. Emerg. Microbes Infect. 9, 1418–1428 (2020). 32529952 10.1080/22221751.2020.1780953 PMC7473193

[27] X. Lei ., Activation and evasion of type I interferon responses by SARS-CoV-2. Nat. Commun. 11, 3810 (2020). 32733001 10.1038/s41467-020-17665-9 PMC7392898

[28] H. Xia ., Evasion of type I interferon by SARS-CoV-2. Cell Rep. 33, 108234 (2020). 32979938 10.1016/j.celrep.2020.108234 PMC7501843

[29] M. Puray-Chavez ., The translational landscape of SARS-CoV-2 and infected cells. bioRxiv. 10.1101/2020.11.03.367516 (Accessed 16 November 2020).

[30] H. Y. Chen ., Infection of HeLa cells by avian infectious bronchitis virus is dependent on cell status. Avian Pathol. 36, 269–274 (2007). 17620171 10.1080/03079450701447291

[31] Y. Chen ., Structure-function analysis of severe acute respiratory syndrome coronavirus RNA cap guanine-N7-methyltransferase. J. Virol. 87, 6296–6305 (2013). 23536667 10.1128/JVI.00061-13 PMC3648086

[32] J. B. Case, A. W. Ashbrook, T. S. Dermody, M. R. Denison, Mutagenesis of S-adenosyl-l-methionine-binding residues in coronavirus nsp14 N7-methyltransferase demonstrates differing requirements for genome translation and resistance to innate immunity. J. Virol. 90, 7248–7256 (2016). 27252528 10.1128/JVI.00542-16 PMC4984653

[33] J. Li, Y. Liu, X. Zhang, Murine coronavirus induces type I interferon in oligodendrocytes through recognition by RIG-I and MDA5. J. Virol. 84, 6472–6482 (2010). 20427526 10.1128/JVI.00016-10 PMC2903279

[34] Y. Hu ., The severe acute respiratory syndrome coronavirus nucleocapsid inhibits type I interferon production by interfering with TRIM25-mediated RIG-I ubiquitination. J. Virol. 91, e02143-16 (2017). 28148787 10.1128/JVI.02143-16 PMC5375661

[35] E. Mantlo, N. Bukreyeva, J. Maruyama, S. Paessler, C. Huang, Antiviral activities of type I interferons to SARS-CoV-2 infection. Antiviral Res. 179, 104811 (2020). 32360182 10.1016/j.antiviral.2020.104811 PMC7188648

[36] L. Miorin ., SARS-CoV-2 Orf6 hijacks Nup98 to block STAT nuclear import and antagonize interferon signaling. Proc. Natl. Acad. Sci. U.S.A. 117, 28344–28354 (2020). 33097660 10.1073/pnas.2016650117 PMC7668094

[37] S. A. Kopecky-Bromberg, L. Martínez-Sobrido, M. Frieman, R. A. Baric, P. Palese, Severe acute respiratory syndrome coronavirus open reading frame (ORF) 3b, ORF 6, and nucleocapsid proteins function as interferon antagonists. J. Virol. 81, 548–557 (2007). 17108024 10.1128/JVI.01782-06 PMC1797484

[38] M. Frieman ., Severe acute respiratory syndrome coronavirus ORF6 antagonizes STAT1 function by sequestering nuclear import factors on the rough endoplasmic reticulum/Golgi membrane. J. Virol. 81, 9812–9824 (2007). 17596301 10.1128/JVI.01012-07 PMC2045396

[39] M. G. Wathelet, M. Orr, M. B. Frieman, R. S. Baric, Severe acute respiratory syndrome coronavirus evades antiviral signaling: Role of nsp1 and rational design of an attenuated strain. J. Virol. 81, 11620–11633 (2007). 17715225 10.1128/JVI.00702-07 PMC2168762

[40] J. B. Case ., Murine hepatitis virus nsp14 exoribonuclease activity is required for resistance to innate immunity. J. Virol. 92, e01531-17 (2017). 29046453 10.1128/JVI.01531-17 PMC5730787

[41] K. Bidet, D. Dadlani, M. A. Garcia-Blanco, G3BP1, G3BP2 and CAPRIN1 are required for translation of interferon stimulated mRNAs and are targeted by a dengue virus non-coding RNA. PLoS Pathog. 10, e1004242 (2014). 24992036 10.1371/journal.ppat.1004242 PMC4081823

[42] A. L. Totura, R. S. Baric, SARS coronavirus pathogenesis: Host innate immune responses and viral antagonism of interferon. Curr. Opin. Virol. 2, 264–275 (2012). 22572391 10.1016/j.coviro.2012.04.004 PMC7102726

[43] T. Tanaka, W. Kamitani, M. L. DeDiego, L. Enjuanes, Y. Matsuura, Severe acute respiratory syndrome coronavirus nsp1 facilitates efficient propagation in cells through a specific translational shutoff of host mRNA. J. Virol. 86, 11128–11137 (2012). 22855488 10.1128/JVI.01700-12 PMC3457165

[44] X. Wang, E. R. Hinson, P. Cresswell, The interferon-inducible protein viperin inhibits influenza virus release by perturbing lipid rafts. Cell Host Microbe 2, 96–105 (2007). 18005724 10.1016/j.chom.2007.06.009

[45] J. C. C. Hsu, D. W. Reid, A. M. Hoffman, D. Sarkar, C. V. Nicchitta, Oncoprotein AEG-1 is an endoplasmic reticulum RNA-binding protein whose interactome is enriched in organelle resident protein-encoding mRNAs. RNA 24, 688–703 (2018). 29438049 10.1261/rna.063313.117 PMC5900566

