# Supplementary material for: Translational shutdown and evasion of the innate immune response by SARS-CoV-2 NSP14 protein
Source: Proc Natl Acad Sci U S A. 2021 May 27;118(24):e2101161118. doi: 10.1073/pnas.2101161118 (PMC8214666; doi:10.1073/pnas.2101161118)
Supplement: Supplementary File [file pnas.2101161118.sapp.pdf]

PNAS
www.pnas.org

Supplementary Information for
Translational shutdown and evasion of the innate immune response by SARS-CoV-2 NSP14 protein

Jack Chun-Chieh Hsu[1], Maudry Laurent-Rolle[1,2], Joanna B. Pawlak[1,2], Craig B. Wilen[1,3,4] and Peter Cresswell[1,5,*]

[1]Department of Immunobiology, Yale University School of Medicine, New Haven, CT 06520, USA.

[2]Section of Infectious Diseases, Department of Internal Medicine, Yale University School of Medicine, New Haven, CT 06520, USA.

[3]Department of Laboratory Medicine, Yale School of Medicine, New Haven, CT 06520, USA

[4]Yale Cancer Center, Yale School of Medicine, New Haven, CT 06520, USA

[5]Department of Cell Biology, Yale University School of Medicine, New Haven, CT 06520, USA.

* Peter Cresswell

Email: peter.cresswell@yale.edu

This PDF file includes:

Figures S1 to S6
SI References

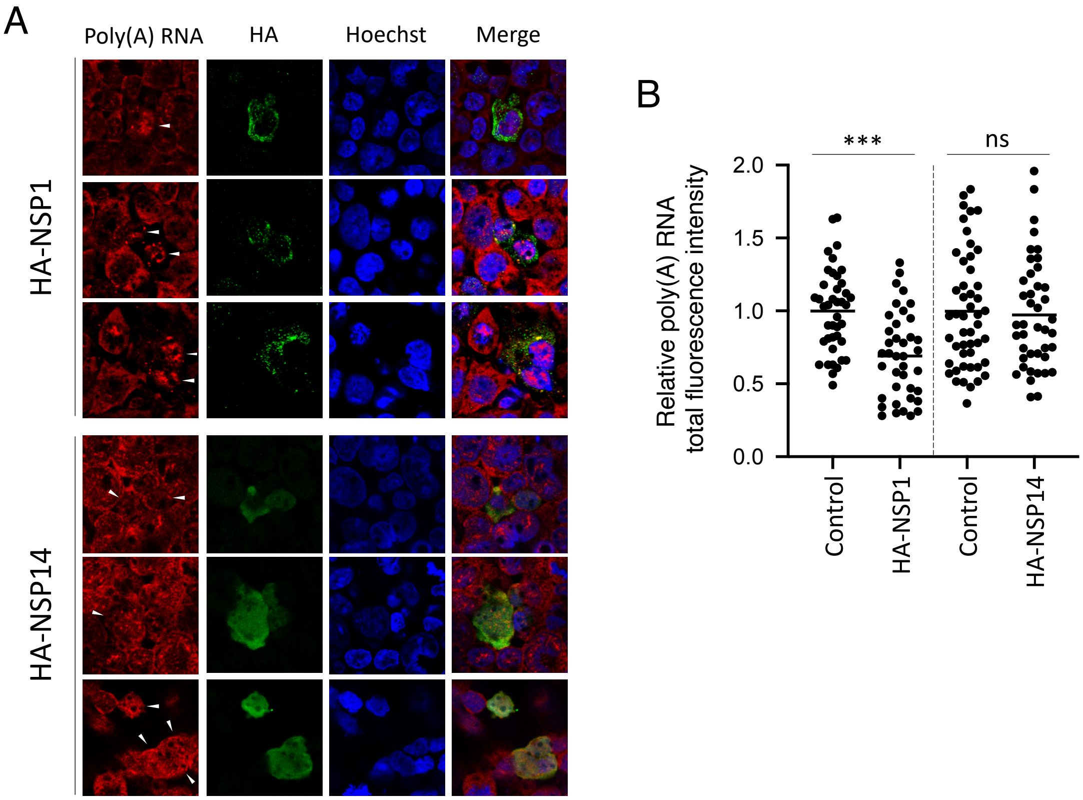

Fig. S1 NSP1 but not NSP14 inhibits poly(A) RNA nuclear export and total poly(A) RNA level.
(A) Representative confocal images of RNA-FISH for poly(A) RNA detection. 293T cells were transfected with plasmids encoding indicated HA-tagged viral proteins for 24 h. Cells were fixed, stained by oligo(dT)-Cy5 probes to detect poly(A) RNA, anti-HA antibody for viral proteins, and Hoechst dye for the nucleus, and analyzed by confocal microscopy. White arrowheads show examples for transfected cells. Images represent > 40 HA-positive cells for each condition. Data represent two independent experiments.
(B) Fluorescence intensity of total poly(A) RNA in individual cells is shown for the indicated experimental condition. Data represent two independent experiments. ***$p < 0.001$ by unpaired Student's t test. ns, not significant.

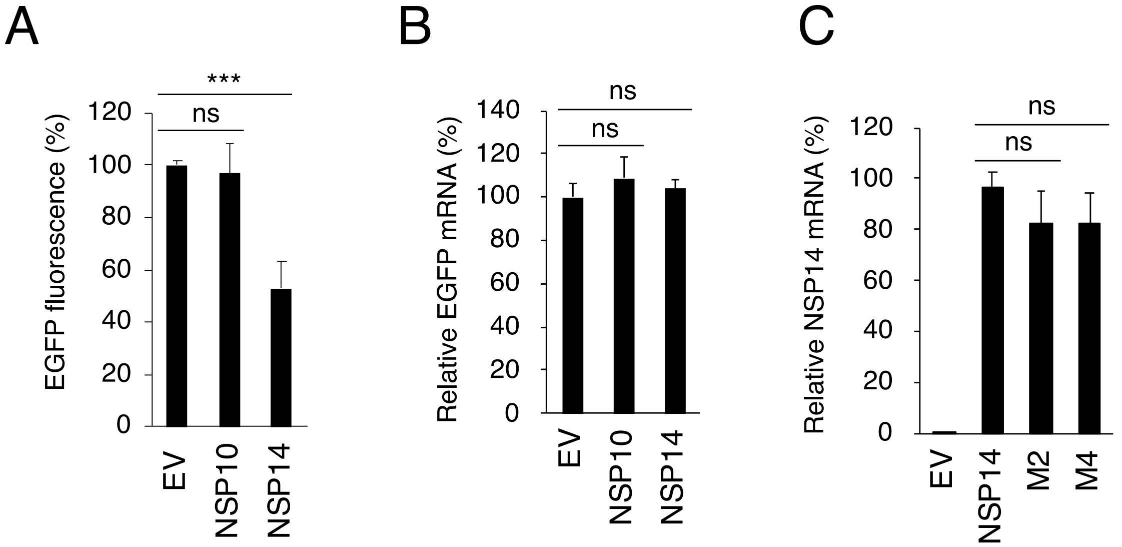

Fig. S2 NSP14 inhibits EGFP protein but not mRNA expression.
(A-B) 293T cells were co-transfected with plasmids encoding EGFP and the indicated viral proteins for 24 h. (A) EGFP fluorescence was monitored by FACS. (B) Relative *EGFP* mRNA was analyzed by RT-qPCR. (C) 293T cells were transfected with plasmids encoding NSP14 or the indicated mutants (M2 and M4) for 24 h. Relative *NSP14* mRNA was analyzed by RT-qPCR.

For (A) to (C), Data are shown as mean ± SD of three biological repeats. ***$p < 0.001$ by unpaired Student's t test. ns, not significant.

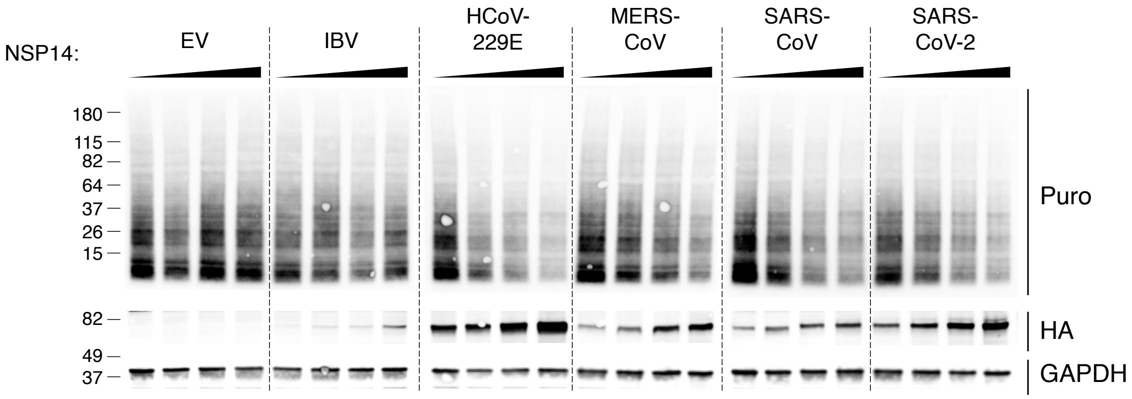

Fig. S3 Coronavirus NSP14 inhibits protein translation.
293T cells were transfected with increasing amounts of plasmid DNA encoding HA-tagged NSP14 proteins from different coronaviruses. After 24 h, cells were puromycin-labeled for 15 min. Puromycin incorporation was determined by immunoblotting using anti-puromycin antibody (Puro). HA-tagged NSP14 proteins were detected by anti-HA antibody (HA). EV, empty vector.

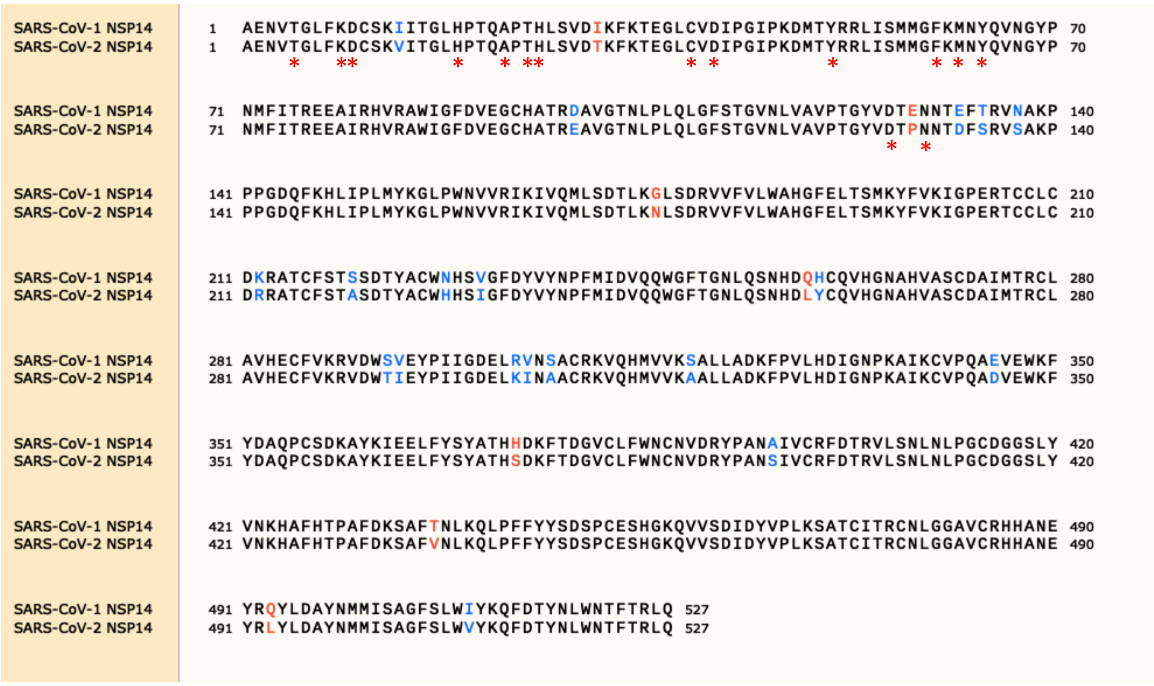

Fig. S4 Primary sequence alignment of SARS-CoV and SARS-CoV-2 NSP14 protein sequences using SnapGene. The protein sequences share 95% sequence identity and 99% sequence similarity. Similar and dissimilar residues are highlighted in blue and red, respectively. Residues at the NSP10-NSP14 interface are highlighted by red asterisks. Sequences of NSP14 in SARS-CoV (accession number ADC35510) and SARS-CoV-2 (QNQ17295) were used for the analysis.

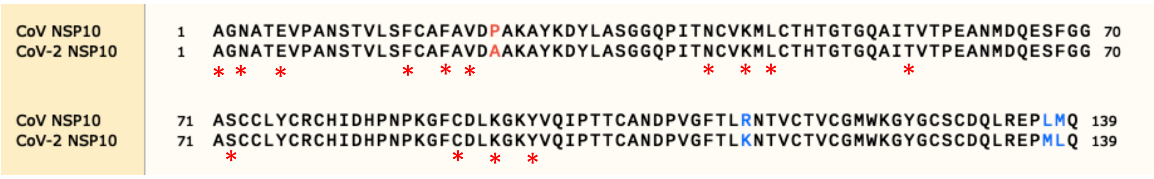

Fig. S5 Primary sequence alignment of SARS-CoV and SARS-CoV-2 NSP10 protein sequences using SnapGene. The protein sequences share 97% sequence identity and 99% sequence similarity. Similar and dissimilar residues are highlighted in blue and red, respectively. Residues at the NSP10-NSP14 interface are highlighted by red asterisks. Sequences of NSP10 in SARS-CoV (accession number ADC35510) and SARS-CoV-2 (QNQ17295) were used for the analysis.

A

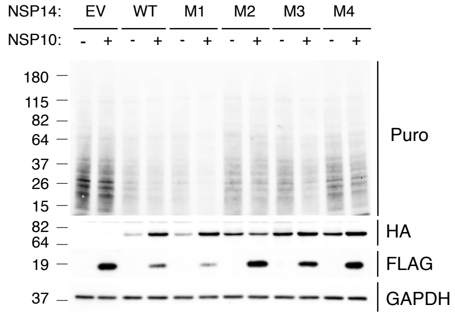

B

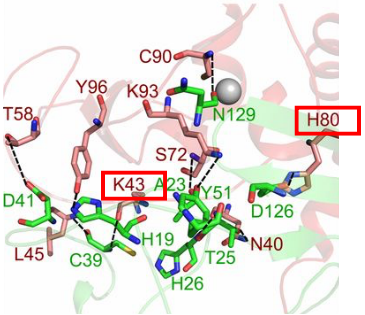

C

Mutation	Interaction (%)	ExoN activity (%)	Viral replication
K43A	38	62	Crippled (reduced by 1 log)
H80A	0	26	Non-viable

D

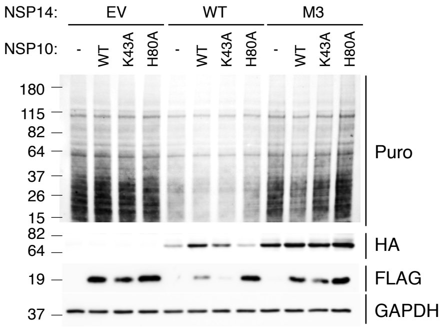

Fig. S6 NSP10 mutants that disrupt the NSP10-NSP14 interaction fail to enhance the translation inhibition activity of NSP14.
(A) 293T cells were co-transfected with FLAG-tagged NSP10 and HA-tagged NSP14 or its mutants for 24 h and puromycin-labeled for 15 min. Puromycin incorporation was determined by immunoblotting. EV, empty vector.
(B) SARS-CoV NSP10-NSP14 interaction interface. NSP10 (red) interacts with the N-terminal ExoN domain of NSP14 (green). K43 and H80 are highlighted in red boxes. Zinc ion is represented as gray spheres. Figure adapted from (1).
(C) Effects of SARS-CoV NSP10 mutations on the NSP10-NSP14 interaction and on the ExoN activity of NSP14 (2). Effects of mutations on the replication of SARS-CoV were determined by plaque assay at 24 hours post infection (2).
(D) 293T cells were co-transfected with FLAG-tagged NSP10 and HA-tagged NSP14NSP14 or their mutants for 24 h and puromycin-labeled for 15 min. Puromycin incorporation was determined by immunoblotting.

SI References

1. Y. Ma *et al.*, Structural basis and functional analysis of the SARS coronavirus nsp14-nsp10 complex. *Proc Natl Acad Sci U S A* **112**, 9436-9441 (2015).
2. M. Bouvet *et al.*, Coronavirus Nsp10, a critical co-factor for activation of multiple replicative enzymes. *J Biol Chem* **289**, 25783-25796 (2014).
